# Pedestrian Origin–Destination Estimation Based on Multi-Camera Person Re-Identification

**DOI:** 10.3390/s22197429

**Published:** 2022-09-30

**Authors:** Yan Li, Majid Sarvi, Kourosh Khoshelham, Yuyang Zhang, Yazhen Jiang

**Affiliations:** 1 Department of Infrastructure Engineering, University of Melbourne, Melbourne, VIC 3010, Australia; 2 State Key Laboratory of Resources and Environment Information System, Institute of Geographic Sciences and Natural Resources Research, Chinese Academy of Sciences, Beijing 100101, China; 3 Department of Urban Planning and Landscape, North China University of Technology, Beijing 100144, China; 4 Department of Urban Planning, Tsinghua University, Beijing 100084, China

**Keywords:** pedestrian origin–destination estimation, multi-view video surveillance, pedestrian trajectories, person re-identification

## Abstract

Pedestrian origin–destination (O–D) estimates that record traffic flows between origins and destinations, are essential for the management of pedestrian facilities including pedestrian flow simulation in the planning phase and crowd control in the operation phase. However, current O–D data collection techniques such as surveys, mobile sensing using GPS, Wi-Fi, and Bluetooth, and smart card data have the disadvantage that they are either time consuming and costly, or cannot provide complete O–D information for pedestrian facilities without entrances and exits or pedestrian flow inside the facilities. Due to the full coverage of CCTV cameras and the huge potential of image processing techniques, we address the challenges of pedestrian O–D estimation and propose an image-based O–D estimation framework. By identifying the same person in disjoint camera views, the O–D trajectory of each identity can be accurately generated. Then, state-of-the-art deep neural networks (DNNs) for person re-ID at different congestion levels were compared and improved. Finally, an O–D matrix based on trajectories was generated and the resident time was calculated, which provides recommendations for pedestrian facility improvement. The factors that affect the accuracy of the framework are discussed in this paper, which we believe could provide new insights and stimulate further research into the application of the Internet of cameras to intelligent transport infrastructure management.

## 1. Introduction

Understanding pedestrian flow characteristics is important for the management of transport infrastructure such as transportation hubs, airports, etc. At the planning and design phase of pedestrian facilities, pedestrian O–D matrices are the main inputs for microscopic pedestrian simulation models that help to identify the bottleneck of pedestrian infrastructure and evaluate alternative designs [1]. At the operation stage, pedestrian O–D and travel time estimation indicate real-time travel demands [2] and can be applied for pedestrian destination prediction [3].

There are four major methods to collect pedestrian O–D traffic data: surveys, mobile sensing, smart card data, and video analysis [4]. Surveys [5] are traditional pedestrian O–D acquisition methods, mainly through on-site investigation or distributed via the Internet, which are less difficult to implement but have the disadvantages of being costly, incomplete and static; mobile sensors such as global positioning system (GPS), Wi-Fi, and Bluetooth are used to obtain the users’ locations and reconstruct their trajectories due to the rapid development of information and communications technology [6,7,8,9,10]. While this captures large amounts of high-frequency data, it is subject to the users’ privacy concerns and reluctance to share their location; smart card data recorded by automatic fare collection (AFC) systems of passengers entering and exiting train stations or bus stops are widely used to analyse passenger travel patterns in urban rail and bus networks [11,12], but these data are not suitable for estimating O–D information for pedestrian facilities without entrances and exits or pedestrian flow inside the facilities. Ubiquitous cameras overcome many of these drawbacks. Meanwhile, due to the huge success of deep learning methods in image understanding, even better than humans, camera-based and deep learning analytics have been used widely for pedestrian location and trajectory data collection [13,14,15]. On a microscopic level, pedestrian detection and tracking techniques are applied to track individuals and collect trajectories, from which pedestrian O–D matrices can be generated [13,15,16]. On a macroscopic level, overall crowd motion information, for example, the crowd sources (potential origins) and sinks (potential destination) can be extracted by optical flow models, where the crowds are treated as a set of moving particles, from which the particle trajectories are generated and clustered [14,17].

Although many image-based methods and applications have been developed in recent years, there is still a gap in the current research on pedestrian O–D estimation across multiple disjoint cameras. While multiple cameras are usually necessary to have complete coverage in large transport hubs, existing methods lack the ability to reconstruct a complete trajectory across multiple views. Specifically, when the tracking fails in one camera view, it is challenging to re-identify the pedestrian in another view and reconstruct the complete trajectory.

To fill in this gap, we propose a novel pedestrian O–D estimation framework and address the challenge from three aspects:

(1) We develop and test a novel pedestrian O–D estimation framework based on multi-camera pedestrian re-identification (re-ID). By identifying the same person in different camera views, the O–D of each identity can be generated.
(2) We compare the state-of-the-art deep neural networks for pedestrian re-ID at different congestion levels and improve the accuracy in crowded scenarios by employing data augmentation techniques.
(3) We generate an O–D matrix based on the trajectories and calculate the resident time, which provides recommendations for pedestrian facility improvement.

The rest of the article is organised as follows. Section 2 describes the related works on pedestrian O–D estimation and pedestrian re-ID. Section 3 explains the details of the proposed framework. Section 4 evaluates the framework and conducts a preliminary O–D analysis based on the framework. The last section draws our conclusions and proposes future directions of this research.

## 2. Related Work

Pedestrian O–D estimation is mainly based on trajectories. *Pedestrian tracking* for trajectory estimation in single camera views has been a topic of intensive research in computer vision. The authors in [13] discussed the methods of collecting pedestrian trajectories under laboratory settings where overhead cameras perpendicular to the floor allow for a view without occlusion. Ref [15] localised pedestrians of small groups in the real environment for analysing pedestrian walking behaviour. Due to the insufficiency of single view tracking in large-scale pedestrian facilities, pedestrian tracking across disjoint cameras have developed to an independent topic *pedestrian re-identification* (re-ID). Unlike *pedestrian tracking*, the key challenge in pedestrian re-ID is to match two images of a person without spatial and temporal correlations but with intensive appearance changes such as lighting, pose, and viewpoint.

Generally, pedestrian re-ID is composed of two main procedures: first, to generate unique person descriptors, and second to compare the descriptors of a *query* with one or two images in the *gallery* to infer either a match or a non-match. Descriptors are descriptions of the visual features of the pedestrians in images such as shape, colour, texture, and motion information, which are usually in the form of feature vectors. Depending on the categories of descriptors, the pedestrian re-ID system can be divided into hand-crafted systems and deep learning based systems [18]. In hand-crafted systems, colour information is most frequently used, represented by colour histograms, and texture features can be extracted by filter templates. As hand-crafted features are variant in different camera environments, a good distance metric is quite important for matching in hand-crafted systems. The purpose of distance metric learning is to keep all of the feature vectors of the same class closer while vectors of different classes fall apart. The most popular formulation is based on Mahalanobis distance, which calculates the Euclidean distance between feature vectors for generating a pairwise distance matrix between the *query* and each *gallery* image, and then finding the pair with the minimum distance [19].

As deep neural networks (DNN) outperform humans in image recognition tasks, they have been applied to extract accurate and robust person descriptors in pedestrian re-ID. Two types of DNNs have been employed for person re-ID: (1) image classification models; and (2) Siamese models based on pair or triplet comparisons. Under the condition of how many identities (class) are available, a pedestrian re-ID model based on image classification determines the individual identity that the *query* image belongs to. A limitation of this method is that it requires a fairly large number of training images to avoid the over-fitting problem, which can affect the performance of the method. Siamese networks have been proposed to solve the problem of the insufficiency of training samples. A Siamese network is a type of neural network structure that contains two or more identical sub-networks. Pair-wise models take two images as input and two sub-networks are connected with pairwise loss function while triplet models take three images as input and three sub-networks are connected by triplet loss function [20].

To overcome the challenges of pedestrian re-ID including variations in human pose and illumination, occlusion, and background clutter, learning robust features is quite important. Three types of features have been discussed in the literature: global features, local features, and attributes. In general, global features emphasise more on the colour and texture features for all regions. ResNet-50 [21], a residual learning framework, has been proposed to learn global features and more importantly, to ease the training of networks that are substantially deeper than those used previously and gain accuracy from a considerably increased depth. Ref [22] argued that both high- and mid-level global features are relevant for cross-domain instance matching, and proposed a unified framework based on ResNet-50 to fuse the high-level features and middle-level features in the early layers for classification.

Recent studies have focused on improving global representation by leveraging local features extracted from human body parts [23,24,25,26]. The authors in [23] regarded the pedestrian as a sequence of body parts from head to foot, and applied long short-term memory (LSTM) in an end-to-end fashion to take into account the contextual information between body parts, enhancing the discriminative capacity of local feature, which aligns better to a full person. In [24], they proposed a network named part-based convolutional baseline (PCB), which conducts uniform partition on the conv-layer for learning local features. In [25], they handled the body part misalignment problem, that is, body parts are misaligned across human detections due to pose/viewpoint change and unreliable detection, and shows that the part-aligned representation leads to a robust image matching similarity. Unlike previous studies based on bounding box detection [26], adopted human semantic parsing to segment accurate body regions, namely foreground, head, upper-body, lower-body, and shoes, and then used them to exploit local cues for person re-identification. Other research works such as [27,28] developed attention-based models to automatically select more discriminative body parts. In [28], they proposed an end-to-end trainable framework to learn context-aware feature sequences and perform attentive sequence comparison simultaneously with dual attention mechanism, while [27] presented a novel harmonious attention convolutional neural network (HA-CNN) for joint learning of attention selection and feature representations in an end-to-end fashion.

Attributes such as middle-level representations of pedestrians have been popular recently. In [29], the authors proposed a multi-level factorisation net (MLFN), a CNN architecture that factorises the visual appearance of a person into latent discriminative factors at multiple semantic levels without manual annotations. The factors such as gender, carrying, clothing, texture, colour, etc., are computed at different levels of the network corresponding to latent attributes of different semantic levels.

Although many methods have been proposed to tackle the problem of pedestrian re-ID, whether they can handle diverse complicated scenes with diverse densities and be applied for crowd congestion management remains unsolved. To fill this gap, in this study, we evaluated these state-of-the-art methods in diverse congestion levels and improved the performances of the models by data augmentation techniques.

## 3. Materials and Methods

### 3.1. Data

**Figure 1:**
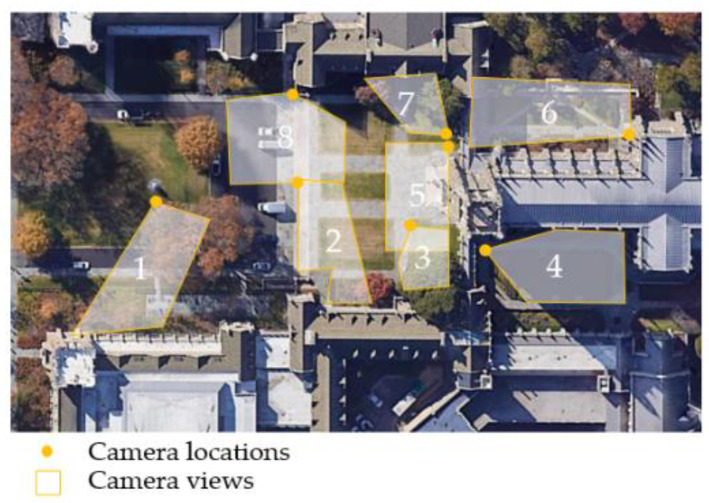
The DukeMTMC dataset with 8 disjoint camera views. Satellite image source: Google Earth.

The dataset used in this study is a public dataset, DukeMTMC-reID [30]. It contains 85-min high-resolution videos recorded with eight cameras on the Duke University campus, as shown in Figure 1. The location was around the Duke University Chapel, an iconic chapel in the centre of the campus (the cruciform roof on the right side of Figure 1). The eight cameras covered all of the sidewalks in front of the chapel to capture the pedestrian flows in and out of the chapel.

In the DukeMTMC dataset, the bounding boxes of pedestrians are provided. The dataset contains cropped pedestrian images from the videos every 120 frames, yielding in total 36,411 bounding boxes with identities. There are 1404 identities appearing in more than two cameras and 408 identities (distractor ID) who appear in only one camera. The DukeMTMC dataset has provided the projective matrices, so we applied the matrices directly to transform the image coordinates of the pedestrian’s feet to world coordinates.

Although the dataset has provided the image frames and bounding box of all pedestrians, which could save our efforts in the first two procedures, in order to test the feasibility of applying the methodology to new videos, we implemented the whole methodology to the raw videos and also proposed new approaches to achieve the first two procedures.

### 3.2. Methods

**Figure 2:**
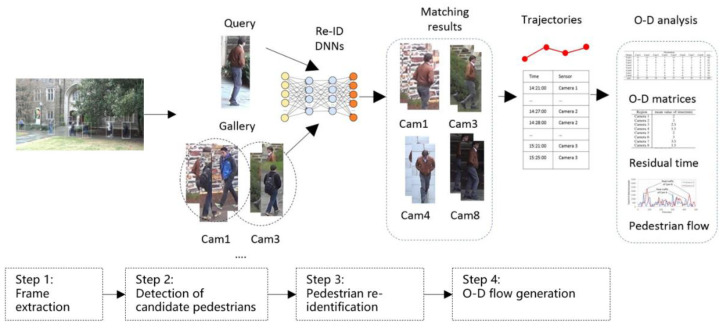
The flow chart of the method. Image source: DukeMTMC dataset.

The proposed framework for pedestrian O–D estimation across multiple disjoint cameras is shown in Figure 2 and described below:

(1) Frame extraction and synchronisation. The image frames named by frame number are extracted from the video footage. Frame synchronisation is to ensure that all of the videos are on the same timeline, so that we can associate the frames of the same time in different camera views.
(2) Detection of candidate pedestrians. This step is a pre-process for pedestrian re-ID. In order to remove the cluttered background, the region-of-interest (ROI) of pedestrians are detected using the deformable part model (DPM) and pedestrian images are cropped from the image frames based on the bounding boxes. All of the pedestrian candidate images are collected in a *gallery*, which would be used for matching.
(3) Pedestrian re-identification (re-ID). The purpose of this step is to classify the detections into different identities. The approach is to initially generate the trajectories in the same camera, calculate the averaged features for each trajectory, and then match the features across different cameras.
(4) Mapping the image coordinates to world coordinates and generating the pedestrian O–D flows. The image coordinates of the pedestrian’s feet are converted to world coordinates to obtain all of the historical locations of the pedestrian. By organising the detection in a chronological order, pedestrian O–D flows can be calculated based on the accumulated trajectories.

More details of each procedure are explained in the following sections.

#### 3.2.1. Frame Extraction and Synchronisation

First, the image frames are extracted from video footage based on a certain frame interval using OpenCV packages. The frame interval was set as 120 in this study, which is equal to 2 s at a frame rate of 60 fps. Then, all of the frames are named after the frame count number using the native package function. Two seconds (i.e., 120 frames) was chosen as the frame interval because of the high computational cost of extracting the pedestrian features from all frames in the video and the performance of the algorithm is degraded due to the presence of a large amount of noise [31]. Key frame extraction is to retain only one feature in a similar feature set and use only some of the discriminative features for recognition to improve the recognition accuracy and efficiency; generally, key frames are frames with different views or different pedestrian postures within the video. In the observation of pedestrian walking posture, it is noted that pedestrians walk with alternating foot movements, which has obvious periodicity, and the time features are embedded in these walking cycles (citations) [32], which indicates that the walking cycle can be used as the smallest unit to divide the video data, which is about changing the walking posture once in 2 s, so the pedestrian features every 2 s must be the most distinct and can be used as key frames.

Since each camera has its own local time starting at frame 1, in order to locate the same frame in different camera views, a master camera is selected, and other cameras are synchronised to the master camera. For example, “frame_1.jpg” in camera 1 is synchronised to “frame_5543.jpg” in camera 5. An alternative approach is that all of the frames can be named after the local time, for example, “frame_20160408_195010.jpg” means that this frame was captured at 19:50:10 on 8 April 2016. If the recording start time of each camera and the frame count number are known, the capture time of each frame can be inferred. Then, it is possible to retrieve the frames at the same time stamp.

#### 3.2.2. Pedestrian Detection and Generation of Gallery Images

The cluttered background in different camera views could cause confusion in the matching process. The general approach is to detect the region-of-interest (ROI) of the pedestrians from the image frames and generate the bounding box of each pedestrian. Then, the pedestrian candidate images can be cropped according to the bounding boxes. All of the candidate images are collected in the *gallery* for person re-ID in the next step.

The deformable part model (DPM) [33] is applied to generate the ROI automatically. The DPM model is very successful in pedestrian detection, which represents objects by component parts arranged in a deformable configuration and is less sensitive to transformations in object pose, viewpoint, and nonrigid deformations.

#### 3.2.3. Pedestrian Re-Identification

1. Data augmentation at different congestion levels

Considering the practical needs of crowd management, we wanted to experiment with images of crowds at different levels of congestion. However, a dataset with such images does not currently exist and we cannot obtain a sufficient number of training images for different congestion levels from one dataset. To overcome this issue, we enlarged the datasets by creating pedestrian images at different congestion levels, often referred to as *data augmentation*. Data augmentation techniques typically include flipping, rotating, scaling, cropping, translating, adding Gaussian noise, or applying generative adversarial networks (GAN) to transform the images. In congested scenes, only the upper body part of the pedestrian is visible, and the lower part is occluded by the surrounding people. Based on this idea, we applied image cropping and cut the lower part of the pedestrian image to simulate the effects of different congestion levels.

**Figure 3:**
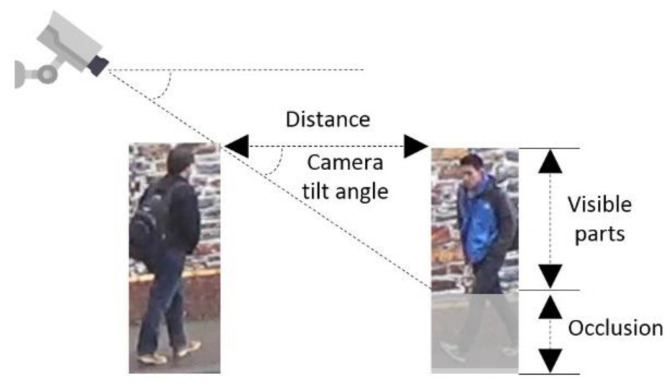
The idea of calculating the percentage of visible body part. Image source: Duke-MTMC dataset.

To determine the percentage of cropping, we approximately calculated the percentage of the visible body at different congestion levels. Level-of-service (LOS) is the most commonly used indicator to measure congestion. According to [34], LOS is defined as the average pedestrian space S (ft^2^/p, square feet per person), as listed in the first column in Table 1. Assuming a rectangular area surrounding the pedestrian, the distance between the pedestrians is the square root of the average pedestrian space. Since the side of the distance and the side of the visible body part form a right-angled triangle, as shown in Figure 3, the percentage of the visible body *p* can be calculated based on Equation (1).

(1) $$p=\frac{\tan\delta \times D}{H}$$

where δ is the camera tilt angle; *D is* the average distance; *H* is the height of the person. Since the height of each pedestrian and camera tilt angle cannot be obtained, in our experiments, we assumed an average pedestrian height of 1.70 m and an approximate tilt angle of 45 degrees. The final calculated value of the image cropping percentage can be seen in Table 1.

With this data augmentation, for each pedestrian image, we generated six new images, each representing one level of congestion, which extend the original dataset and were applied to train the deep neural networks.

2. Deep learning architectures for person re-ID

A Siamese neural network is employed to determine if two or more pedestrian images match and can be assigned the same ID. The network consists of three parts: the first part uses deep neural networks (DNNs) for feature extraction, then the second part performs feature tracking in the single camera view and calculates the average feature of each trajectory, and finally, the loss function is used to match in multiple views.

In the first part, that is, the robust feature extraction, we evaluated and compared three types of DNNs for feature extraction, as described in related work. We chose state-of-the-art ResNet-50 [21], ResNet50-fc512, and ResNet50-mid [22] for the global feature extraction, HA-CNN [27] for local feature extraction, and MLFN [29] for the attribute based models.

Before applying the features for final matching, we performed an in-camera feature merging, which was to generate the trajectories of each ID in single camera views, and then calculated the average features of each trajectory. The advantage of performing this step is that, on one hand, the trajectory-based features have proven to be more robust than the features of a single pedestrian image, while on the other hand, pedestrian re-ID in single cameras can also be used for pedestrian tracking in single camera views, which has shown good performance in the MOT pedestrian tracking benchmark [35]. Based on this work, first, the bounding boxes of adjacent frames were merged into tracklets by the Kuhn–Munkras algorithm in a small window. Then, hierarchical clustering was used to merge the tracklets into trajectories. Finally, the averaged re-ID features of the trajectories were calculated for all of the trajectories in all the cameras.

In the last part, that is, the matching in disjoint camera views, pairwise and triplet loss functions were compared. Cross-entropy was used for the pair-wise network, while triplet loss [20] was used for the triplet network.

#### 3.2.4. O–D Flow Generation

After retrieving all of the matching pedestrians, the location and time information of the pedestrian at the time it was captured need to be extracted. For the location information, the position of the pedestrian in the picture is converted to the position on the planar map by projection transformation using at least three pairs of reference points in the image and the real-world map. Time information can be obtained by naming the frames of this image.

**Figure 4:**
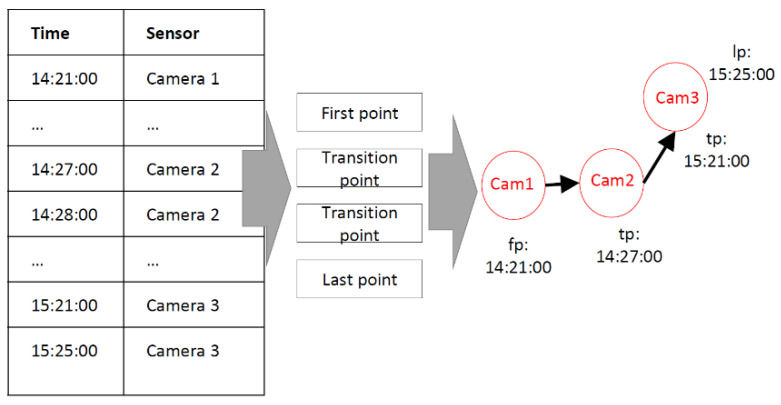
The trajectory data structure of each pedestrian.

After extracting the location and time information of each pedestrian’s observed point, all of the locations are connected chronologically to generate the pedestrian’s trajectory data. In order to perform the following O–D analysis more efficiently, the points were classified as three types including the first point, transition point, and last point, as shown in Figure 4. The point where the pedestrian was identified first was regarded as the first point. The point where the pedestrian was identified last was regarded as the last point. Other points where the pedestrian appeared were treated as transition points.

## 4. Results

### 4.1. Training Strategies

The number of identities in the training and test sets were the same, both were 702. In the testing set, one image for each identity in each camera was selected as the *query* image and the other images were placed in the *gallery*. The training parameters used to train all of the models included the image height and width (256 × 128), optimiser (AMSGrad), learning rate (0.0003), epoch (60), step size (20 40), train batch size (32), and test batch size (100). The input image size and epoch for the HA-CNN were different, which were 160 × 64 and 300 epochs, respectively.

### 4.2. Model Evaluation

**Figure 5:**
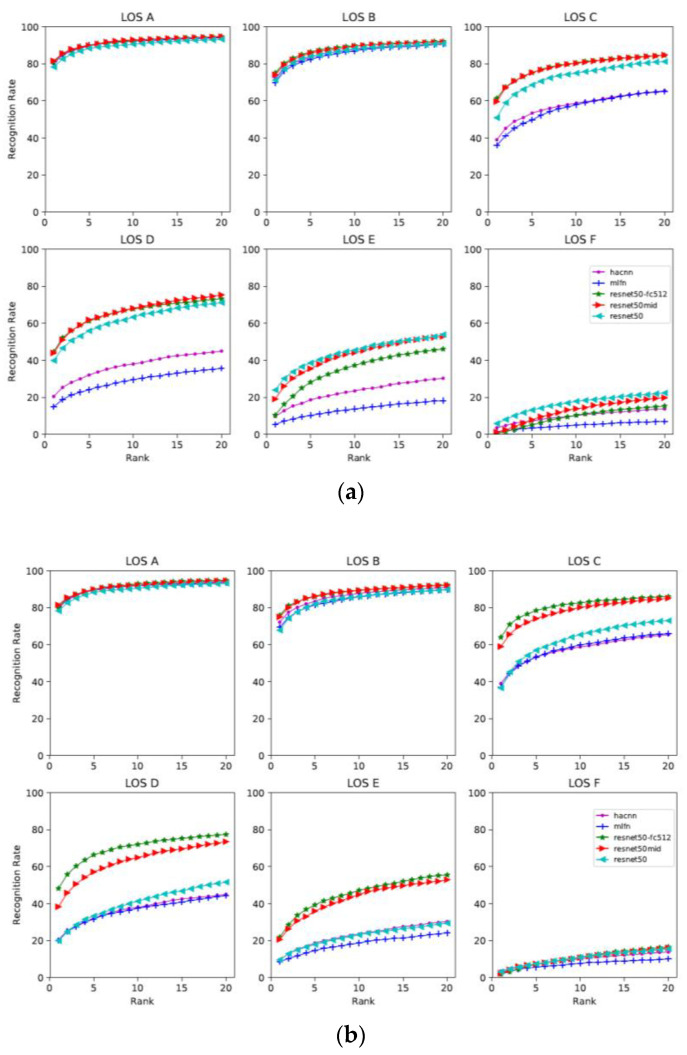
The CMC curve of the image-based methods under different congestion levels. (**a**) Pair-wise network. (**b**) Triplet network.

In order to evaluate the models, we fine-tuned the models following the standard procedures for all models and reported the top-k ranking rate. The top-k ranking rate is equal to the correct matching rate that the right match appears in the top-k predictions. The cumulative matching characteristic (CMC) curve is a precision curve that is typically used to provide detection or recognition precision for each rank, from the top-1 to top-20, as shown in Figure 5.

When evaluating which features are more robust, we found that when the congestion level is low and pedestrians are unobscured (maximum 10% obscured), for example, at LOS A and B, all models showed similar performance and the five curves were close, as shown in Figure 5. However, when the congestion level became higher and the pedestrians were partially occluded (10–30% area occluded), for example, LOS C and D, the performance between the different models began to differ. Overall, the models based on global features such as ResNet50, ResNet-fc512, ResNet50-mid showed better performance than the local feature- and attribute-based models at high LOS (from LOS C to F). This is despite the fact that the local feature-based model HA-CNN and attribute-based model MLFN are specifically designed models for pedestrian re-ID. Therefore, improving the performance of these models in congested scenarios should be one possible direction for future improvements.

The evaluation of loss functions showed that the performance of ResNet50 is unfavourably affected by the triplet loss function. The ResNet-50 model with cross entropy performed better than the model with triplet loss, as shown in Table 2. Conversely, other models with triplet loss showed slightly higher precision than the models with cross entropy.

### 4.3. O–D Flow Estimation

Using the transition points, we could determine where the pedestrians came from and where they went. In order to generate an O–D table, the trajectories between the origin and destination were aggregated. Table 3 presents an O–D table generated using the extracted transition points. We observed that the total inflow and outflow in the surveillance area of camera 2 were relatively high, exceeding 600 trips, while those in camera 4 were relatively low, around 160 trips. The flow between camera 1 and camera 2 was much higher than that between the other cameras, more than 300. From the camera map, we can infer that the road connecting these two camera views is the most frequently used around the chapel.

**Figure 6:**
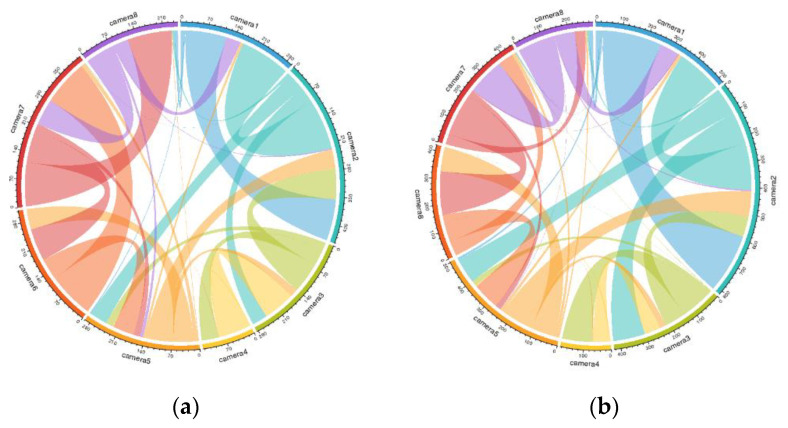
Pedestrian O–D flow in the first half hour (**a**) and the second half (**b**) of the video.

Dynamic O–D flows over different time periods can be visualised using migration flow graphs, as shown in Figure 6. The pedestrians’ origins and destinations are represented by the different coloured segments of circles. The estimated size of the flow is indicated by the width of the link line and can be read by the tick marks on the outside of the circular segments. The direction of the pedestrian flow is determined by the colour of the link and the coding of the colour at the other end. We can see that the flow from camera 1 to camera 2 increased in the second half hour, as indicated by the larger width of the blue arc. From the flow scale around the circle, we can also observe that the total flow within the coverage of camera 2 was the largest, as indicated by the longest arc length. Furthermore, since the red arc length became shorter, the total pedestrian flow in the coverage area of camera 7 reduced in the second half hour, as indicated by the shorter length of the red arc.

## 5. Conclusions

In this study, we proposed a novel pedestrian O–D estimation framework based on multi-camera pedestrian re-identification (re-ID). Combined with the applications in transport infrastructure management, we evaluated and compared the state-of-the-art models of pedestrian re-ID at different congestion levels. The data augmentation technique was applied to improve the accuracy in crowded scenarios. We believe that this work can provide new insights into automated pedestrian flow estimation and stimulate the research on intelligent transport infrastructure management.

In future work, this research will be continued and extended in several ways:

(1) The entire network for multi-view pedestrian re-ID can be designed in an end-to-end fashion. Since the pedestrian detection and the pedestrian re-ID were processed separately in this article, they may share the same backbone for feature extraction, which can make the framework more efficient when processed online. The extracted features can be used not only to generate the bounding box of a pedestrian, but also for subsequent matching.
(2) An additional input channel can be applied, that is, the depth information collected by the depth sensors, and the information integrated into the current structure. The depth image contains information relating to the distance of the surfaces of pedestrians from a viewpoint from which the skeleton information and anthropometric measurements of the pedestrians can be extracted, which have been proven to be more robust than the appearance features extracted from RGB cameras in different camera environments.
(3) State-of-the-art pedestrian re-ID techniques take the bounding boxes of pedestrians as inputs. Since the background is useless in the pedestrian re-id process, image segmentation can be combined with pedestrian detection to generate a more accurate pedestrian shape and completely remove the background.

## Figures and Tables

**Table 1:** The relationship between the LOS and the percentage of visible body parts.

LOS	Avg. Space (S, ft^2^/p)	Avg. Distance (D, Meter)	Visible Body Parts (P)
A	>35	>1.803	Whole body
B	25–35	1.524–1.803	>90% body
C	15–25	1.180–1.524	70–90% body
D	10–15	0.964–1.180	57–70% body
E	5–10	0.682–0.964	40–57% body
F	<5	<0.682	<40% body

**Table 2:** The model performance using pair-wise and triplet networks.

Categories	Methods	LOS A	LOS B	LOS C	LOS D	LOS E	LOS F
Pair-wise network							
Global	ResNet50	78.3	71.2	50.9	39.9	24.0	5.9
	ResNet-fc512	81.0	75.1	61.4	44.6	10.4	0.5
	ResNet50-mid	81.6	74.1	59.6	44.0	19.1	1.3
Local	HA-CNN	80.1	72.2	39.0	20.5	9.7	3.6
Attributes	MLFN	81.1	69.7	36.0	14.9	5.4	1.3
Triplet network							
Global	ResNet50	77.7	67.8	36.6	19.9	9.6	3.2
	ResNet-fc512	80.5	75.9	64.0	48.2	21.8	1.9
	ResNet50-mid	81.5	75.0	59.0	38.3	20.6	2.4
Local	HA-CNN	79.7	72.2	39.0	20.5	9.7	3.6
Attributes	MLFN	80.4	69.4	37.1	20.1	8.6	2.4

**Table 3:** Origin–destination (O–D) table.

		Destination								
		Cam1	Cam2	Cam3	Cam4	Cam5	Cam6	Cam7	Cam8	Sum
Origin	Cam1	0	336	0	0	12	0	4	24	376
	Cam2	310	0	166	0	144	0	2	15	637
	Cam3	0	158	0	160	38	1	2	0	359
	Cam4	0	1	147	0	7	0	0	0	155
	Cam5	20	151	45	6	0	148	23	9	402
	Cam6	0	0	0	0	161	0	174	0	335
	Cam7	4	0	0	0	38	248	0	155	445
	Cam8	113	9	0	0	10	0	213	0	345
	Sum	443	655	358	166	410	396	418	194	3040
